# Supplementary material for: A living vector field reveals constraints on galactose network induction in yeast
Source: Mol Syst Biol. 2017 Jan 1;13(1):908. doi: 10.15252/msb.20167323 (PMC5293160; doi:10.15252/msb.20167323)
Supplement: Supplementary file 1 — Appendix [file MSB-13-908-s001.pdf]

Appendix for

A Living Vector Field Reveals Constraints On Galactose Network Induction In Yeast

Sarah R. Stockwell and Scott A. Rifkin

correspondence to: sarifkin@ucsd.edu

Table of Contents

Appendix Table S1. Population-level GAL induction phenotypes depend on cells' previous nutrient environments	2
Appendix Table S2. Length and variability of lag times until induction	2
Appendix Table S3. Intercell differences explain expression variation at full induction.	2
Appendix Figure S1. Generation of the vectors in the vector fields	3
Appendix Figure S2. Trajectories of two cells on the vector field	4
Appendix Figure S3. Plasmids used to generate fusion proteins	5
Appendix Figure S4. Photobleaching is not a problem	6
Appendix Figure S5. The cell segmentation pipeline	7
Appendix Figure S6. The logic of cell tracking	8
Appendix Figure S7. An example of cell tracking	9
Appendix Figure S8. Alive and dead cells differ in bright field images	10
Appendix Figure S9. Random forest classification of cells as experimental, control, or bad based on fluorescence	11
Appendix Figure S10. Empirical cumulative distributions of induction times	12
Appendix Figure S11. Combined vector fields for the initial three conditions	13
Appendix Figure S12. Population level depiction of galactose network induction for five conditions	14
Appendix Figure S13. Combined vector fields for all five conditions	15

Appendix Table S1. Population-level GAL induction phenotypes depend on cells' previous nutrient environments.

History condition	Initial Gal1p	Initial Gal3p	Induction lag?	Induction pattern
Glycerol or Raffinose	Absent (1)	Low (3)	Short/none (2,5)	Unimodal, graded (2,6)
Reinduction	Low (2)	Absent (4)	Short/none (2)	Unimodal, graded (2)
LTGR	Absent (1)	Absent (3)	Long (2)	Transiently bimodal (2)

LTGR: long-term glucose repression. *Reinduction*: Full induction in galactose, then up to 12 hours in glucose during which Gal1p concentrations decrease but do not fully disappear. *Glycerol/raffinose*: Non-inducing, non-repressing carbon sources in which Gal3p is basally expressed. The presence of a small amount of either Gal1p or Gal3p when cells are transferred to galactose is associated with a short or nonexistent lag and a unimodal, graded induction. *Graded* refers to the bulk of the population gradually increasing its induction level over time. Populations that induce after LTGR are not significantly graded; instead, most cells are either uninduced or highly induced. (1) (Lohr *et al*, 1995); (2) (Zacharioudakis *et al*, 2007); (3) (Bajwa *et al*, 1988); (4) this study, suggested (but not measured) by (Kundu & Peterson, 2010); (5) (Torchia & Hopper, 1986; Kundu & Peterson, 2010); (6) (Ramsey *et al*, 2006)

Appendix Table S2. Length and variability of lag times until induction

	Gal3p (hours)					Gal1p (hours)			
	25%	50%	75%	IQR		25%	50%	75%	IQR
Glycerol	0.5	0.8	1.2	0.7		1.2	1.4	1.7	0.5
Reinduction	1.7	2.2	2.8	1.1		2.0	2.5	3.1	1.1
LTGR	5.4	6.4	7.5	2.1		6.3	7.4	8.5	2.2

Hours after galactose exposure until 25%, 50%, or 75% of inducing cells reached 10% plateau expression level for each inducer, Gal3p and Gal1p (Fig. 2). The IQR is the number of hours between 25% and 75% of cells inducing.

Appendix Table S3. Intercell differences explain expression variation at full induction.

	Gal3p				Gal1p		
	low	R^2	high		low	R^2	high
Glycerol	0.793	0.802	0.810		0.804	0.812	0.821
Reinduction	0.845	0.863	0.876		0.861	0.875	0.885
LTGR	0.794	0.812	0.821		0.836	0.843	0.851
mix: 0.15% glu	0.798	0.817	0.833		0.840	0.866	0.887
mix: 0.3% glu	0.740	0.763	0.786		0.849	0.863	0.875

The fraction of variation in expression levels at full induction explained by cell-specific variation as opposed to random fluctuations. (Point and 95% confidence interval estimates for R^2).

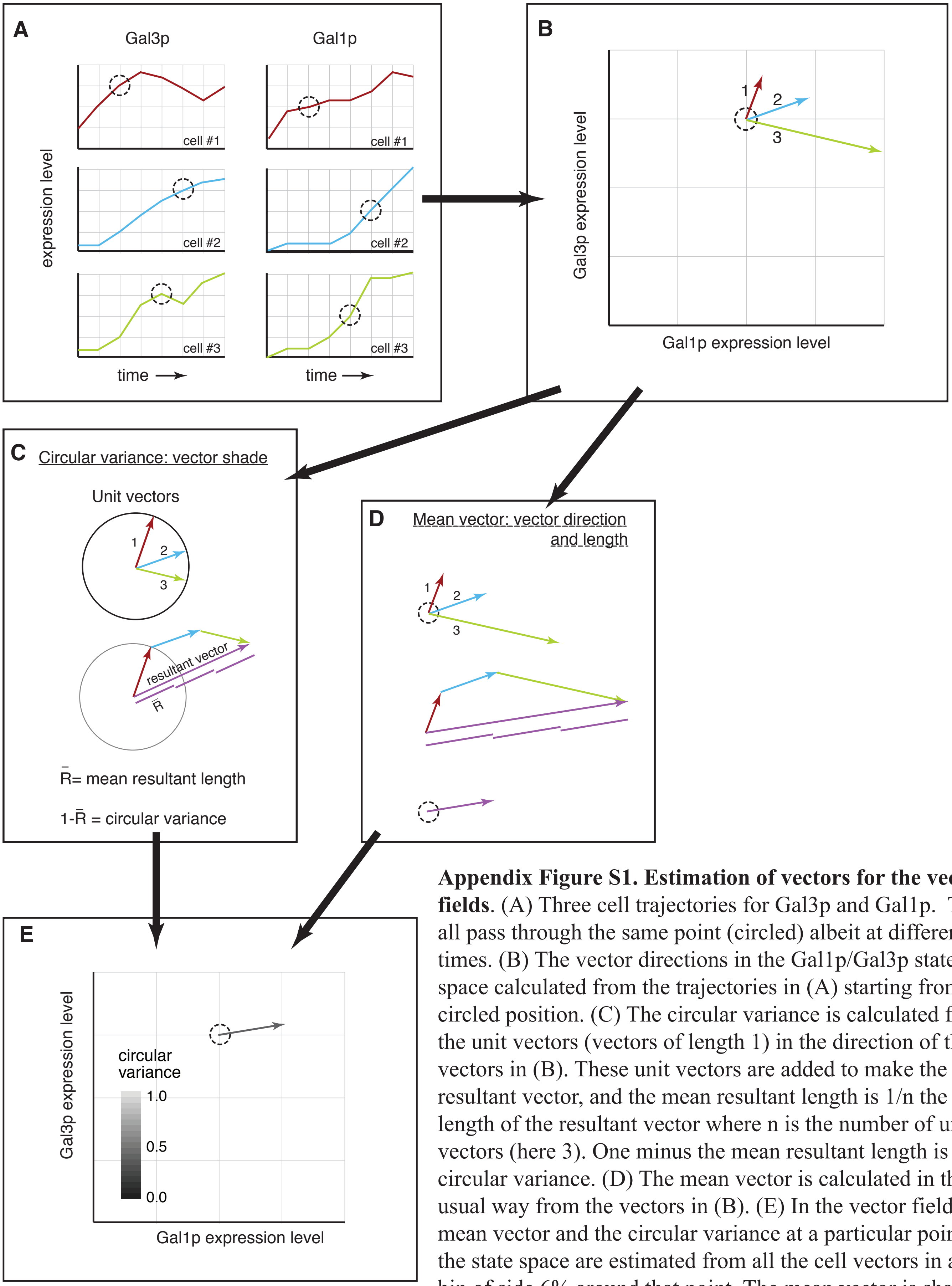

Appendix Figure S1. Estimation of vectors for the vector fields. (A) Three cell trajectories for Gal3p and Gal1p. They all pass through the same point (circled) albeit at different times. (B) The vector directions in the Gal1p/Gal3p state space calculated from the trajectories in (A) starting from the circled position. (C) The circular variance is calculated from the unit vectors (vectors of length 1) in the direction of the vectors in (B). These unit vectors are added to make the resultant vector, and the mean resultant length is 1/n the length of the resultant vector where n is the number of unit vectors (here 3). One minus the mean resultant length is the circular variance. (D) The mean vector is calculated in the usual way from the vectors in (B). (E) In the vector fields, the mean vector and the circular variance at a particular point in the state space are estimated from all the cell vectors in a square bin of side 6% around that point. The mean vector is shaded in greyscale by the circular variance. The vectors in the vector field plots are proportional to the mean vector length; they have been scaled so they do not overlap for visualization.

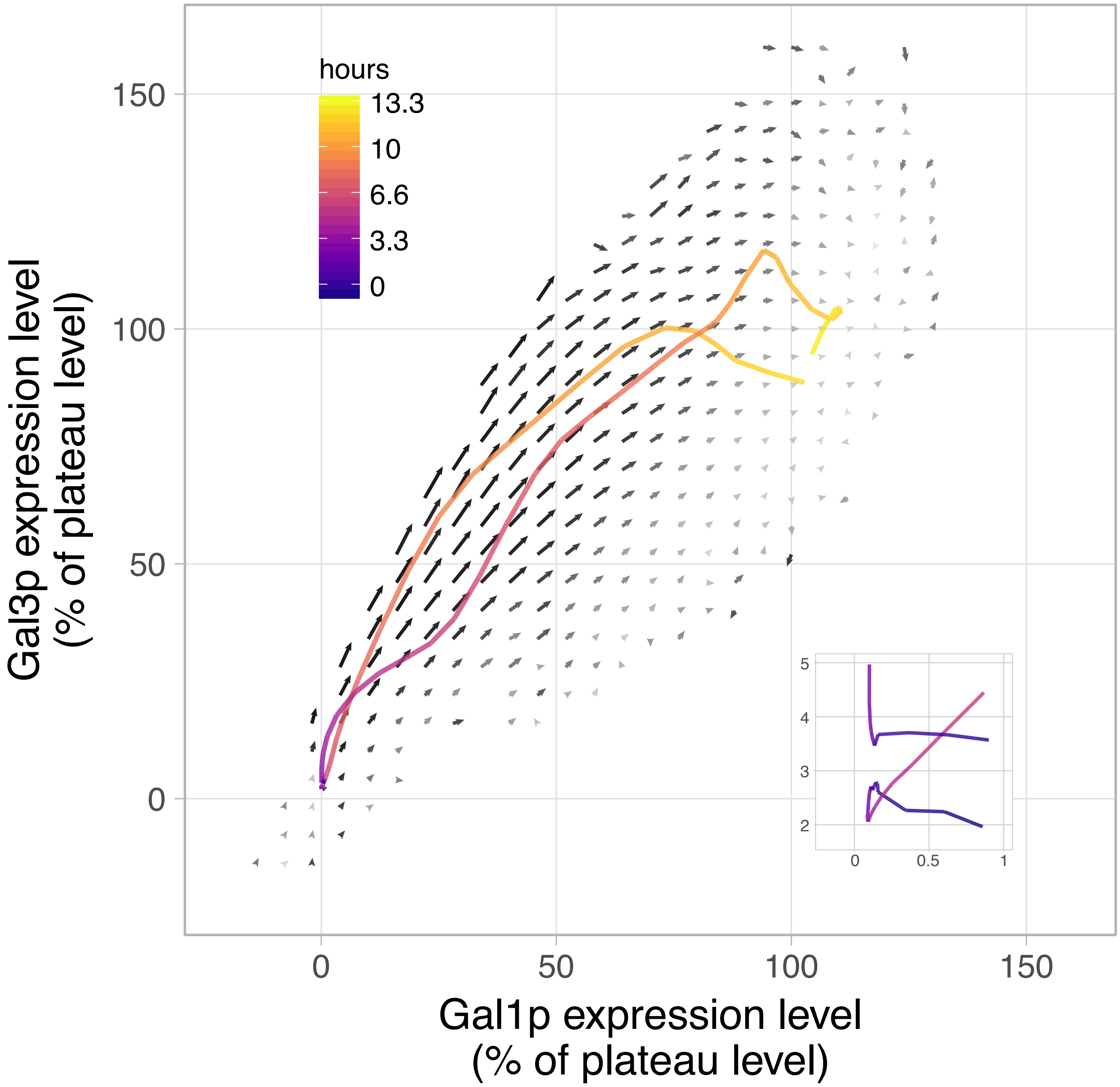

Appendix Figure S2. Individual cell trajectories through the vector field. Trajectories of two cells exposed to galactose following long-term glucose repression in the Ga1p/Gal3p state space. Vector shading is as in Figure 3. The trajectories are color coded by time since galactose exposure. The cell trajectories cross indicating that randomness, other variables, or measurement error play a minor role in determining individual trajectories, even if there is little variation around the average in some regions of the vector field. The inset shows the cells sojourning for several hours in the sticky region near (0%, 0%) before starting to induce.

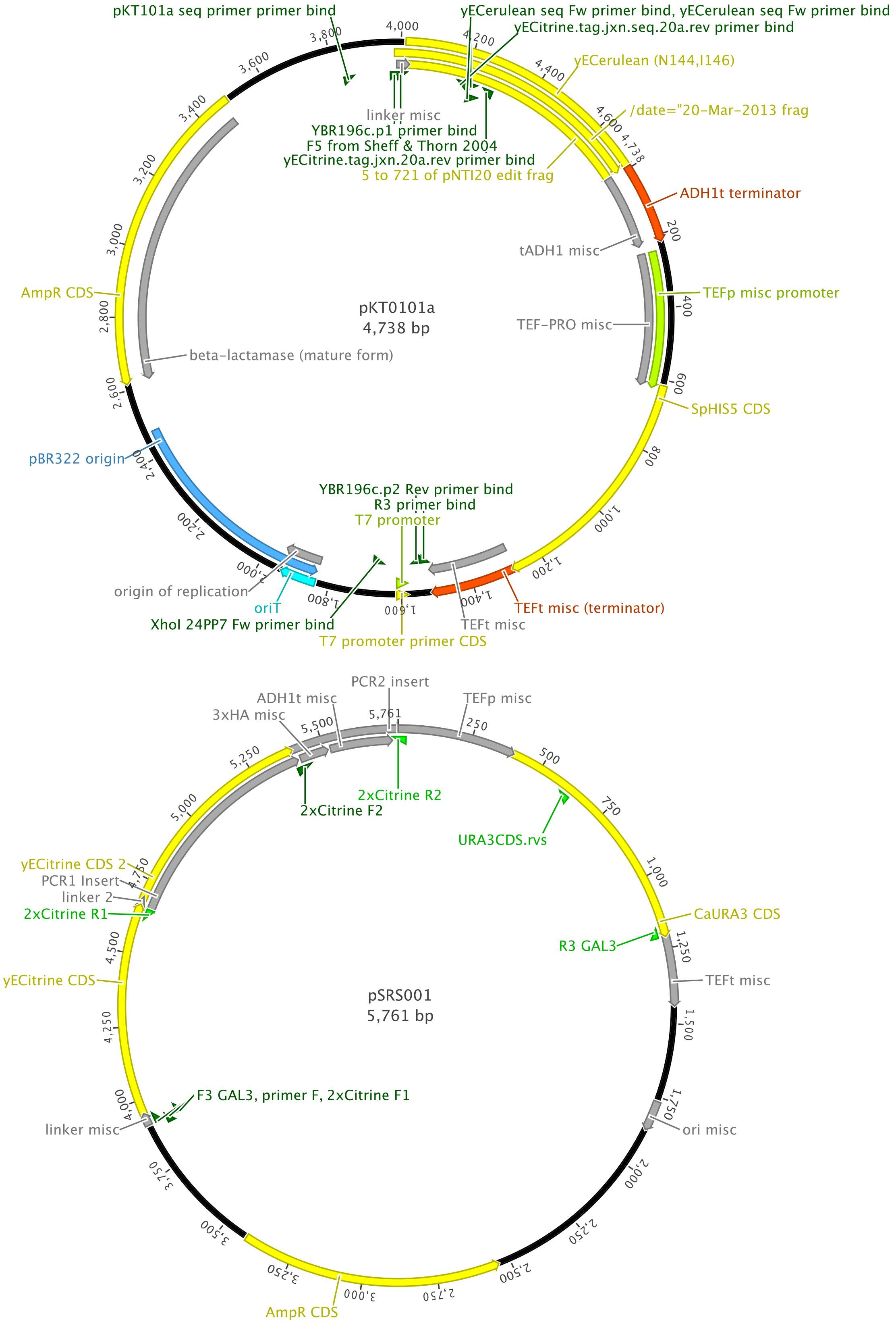

Appendix Figure S3. Plasmids used to generate fusion proteins. pKT0101a was a gift from Natalie Cookson.

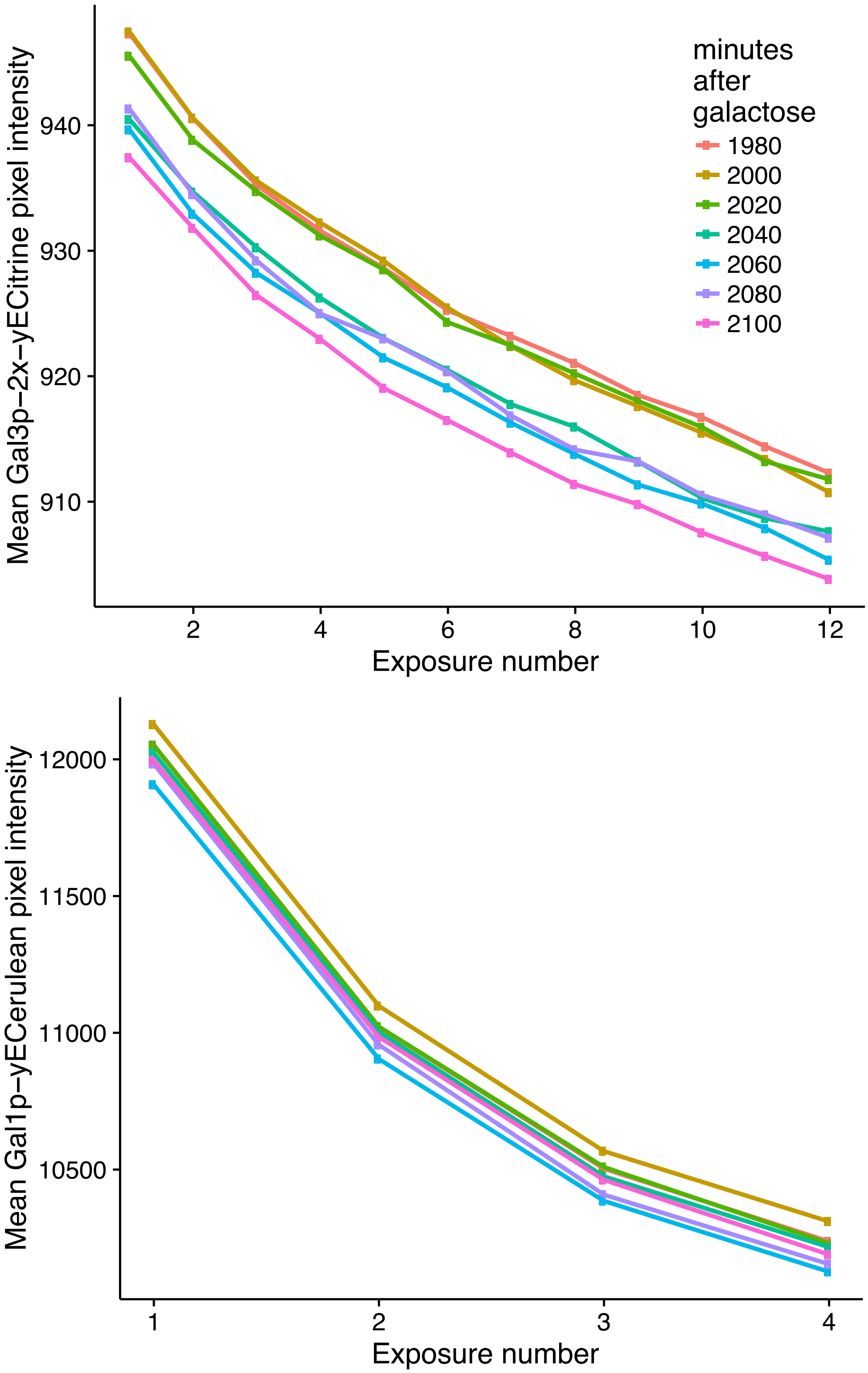

Appendix Figure S4. Photobleaching is not a problem. We took a series of 50-msec fluorescent snapshots at each timepoint. This was needed to capture the full dynamic range of Gal1p-yECerulean but also provided a built-in photobleaching control. Fluorescence levels decline during each series but follow a consistent pattern and recover by the next timepoint 20 minutes later, as shown by the overlapping curves. The curves are mean pixel intensities of seven consecutive frames around 33 hours after galactose exposure in the reinduction condition.

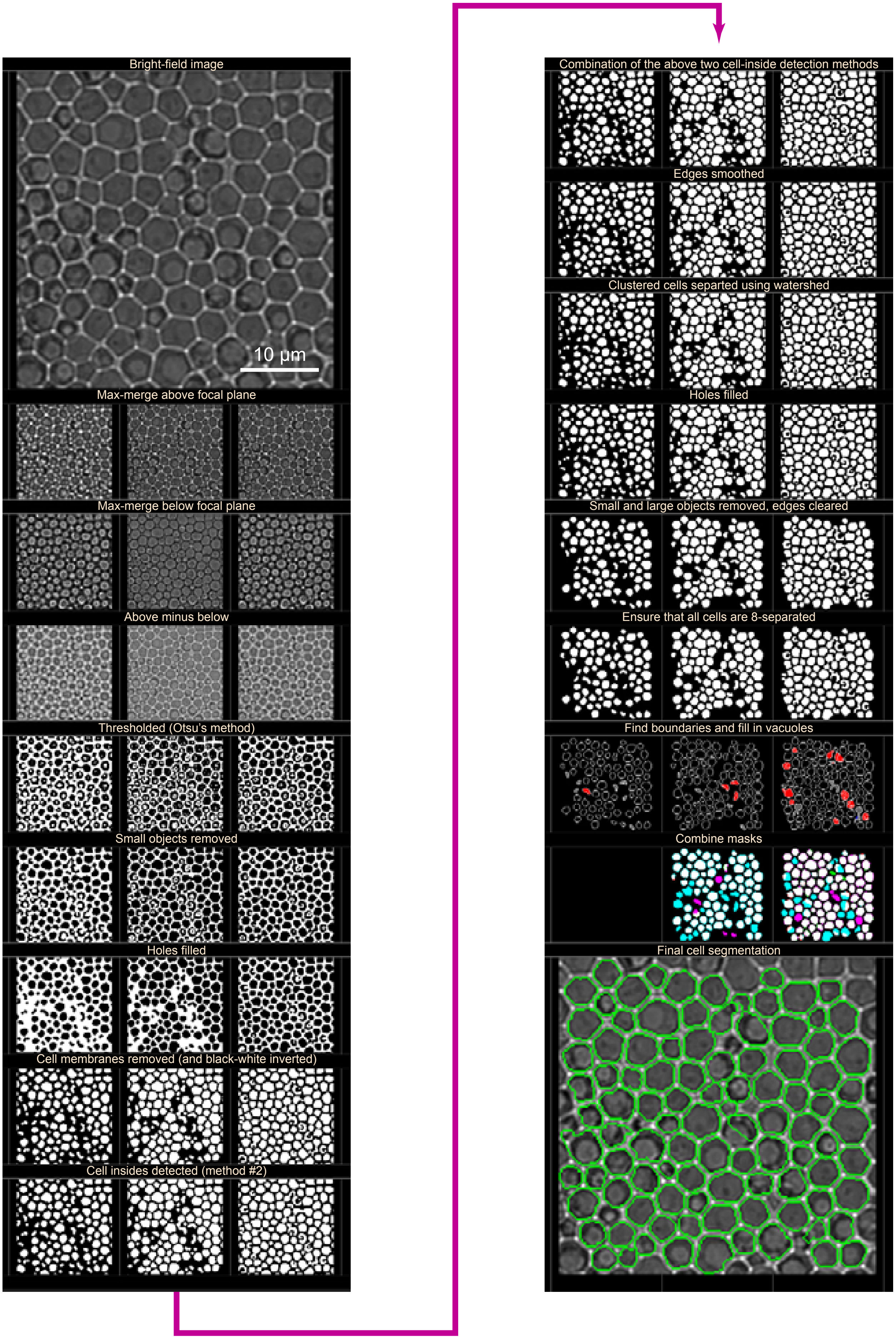

Appendix Figure S5. The cell segmentation pipeline starting from a set of above and below focal plane bright-field images.

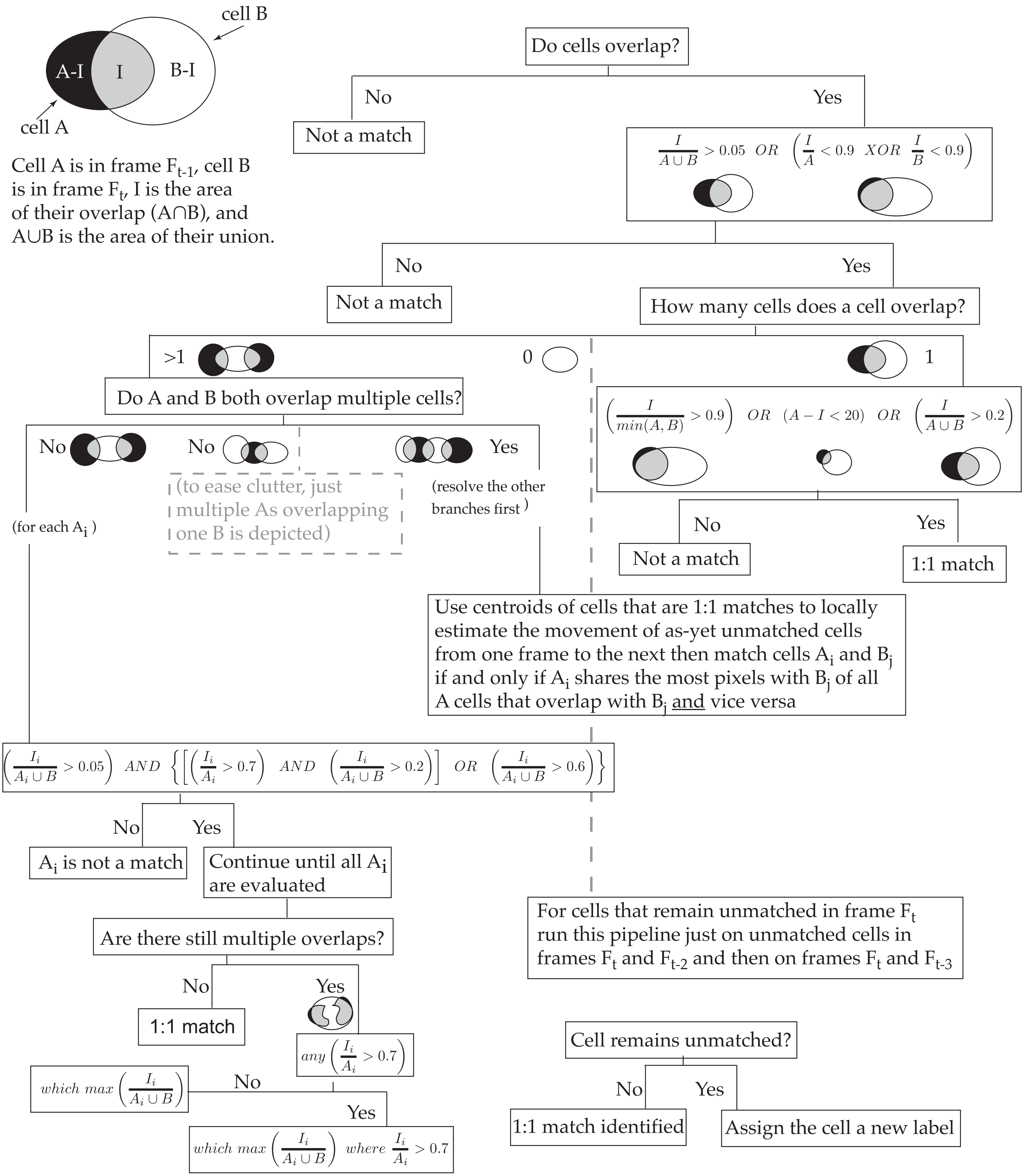

Appendix Figure S6. A flow diagram describing the logic of cell tracking

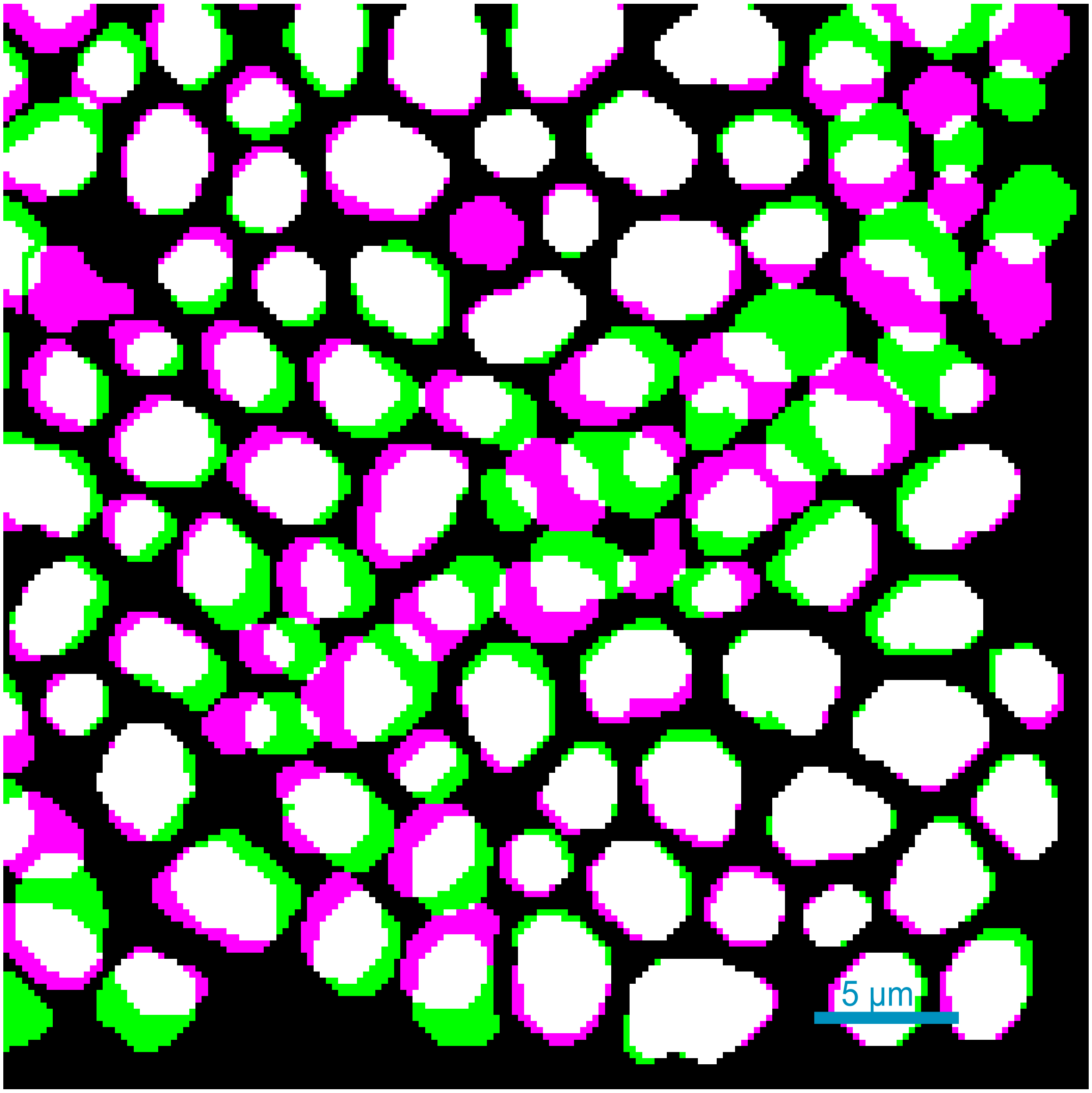

Appendix Figure S7. An example of cell tracking. The green and magenta indicate two successive segmented frames. White represents regions of overlap. This field of view contains many of the kinds of assignment situations resolved by the tracking logic (Appendix Figure S6). The scale bar is 5 μm long.

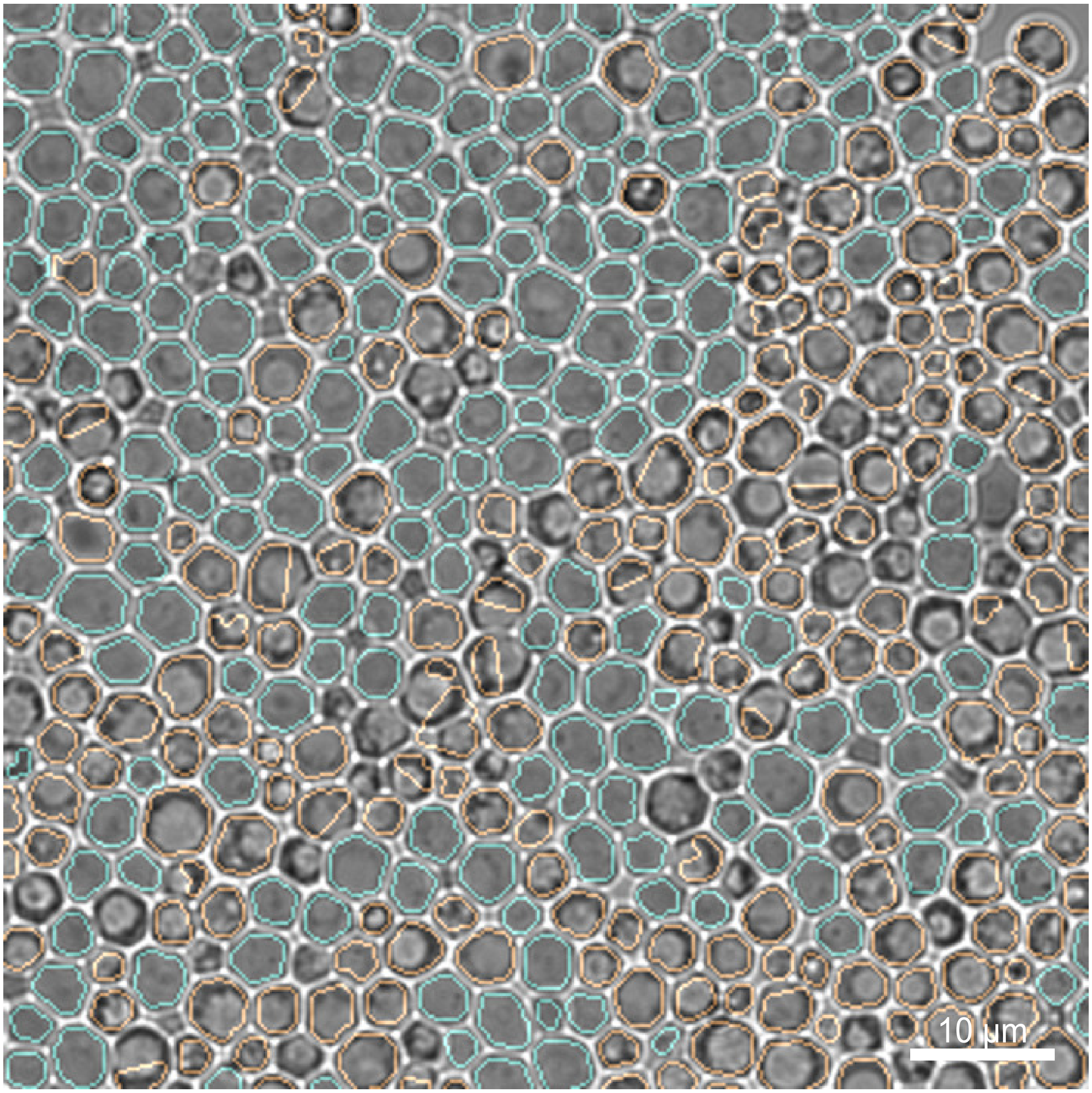

Appendix Figure S8. Alive and dead cells are noticeably different in bright field images. Random forest classification of cells as alive (cyan outlines) or dead (brown outlines) in an glucose history image around 6 hours after galactose exposure. The scale bar is 10 µm long.

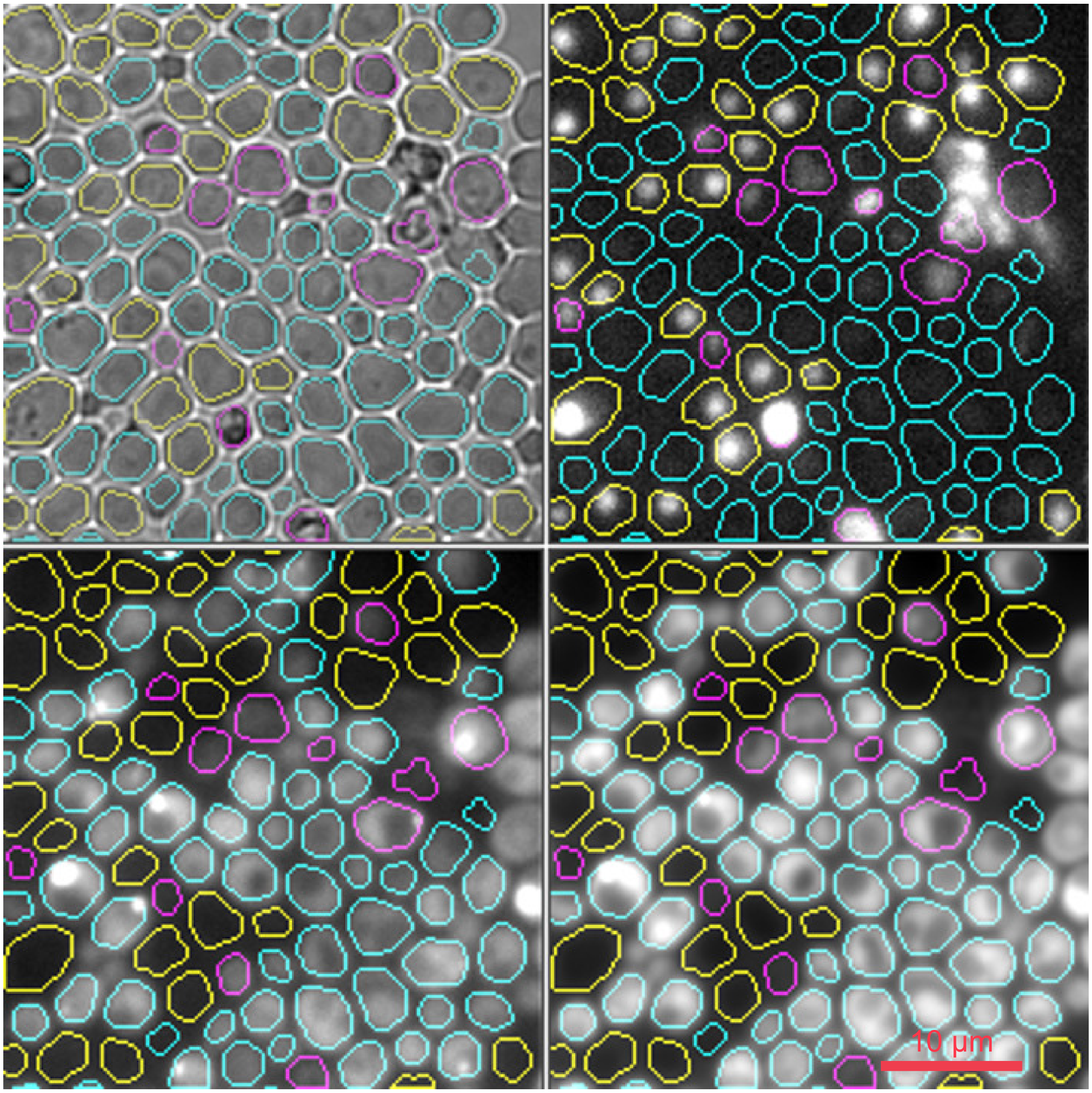

Appendix Figure S9. Random forest classification of cells as experimental, control, or bad based on fluorescence. The images are (clockwise from the upper left): bright-field, mCherry, 2x-yECitrine, yECeruelan. Cyan outlines indicate experimental cells, yellow indicate control, and magenta indicate bad.

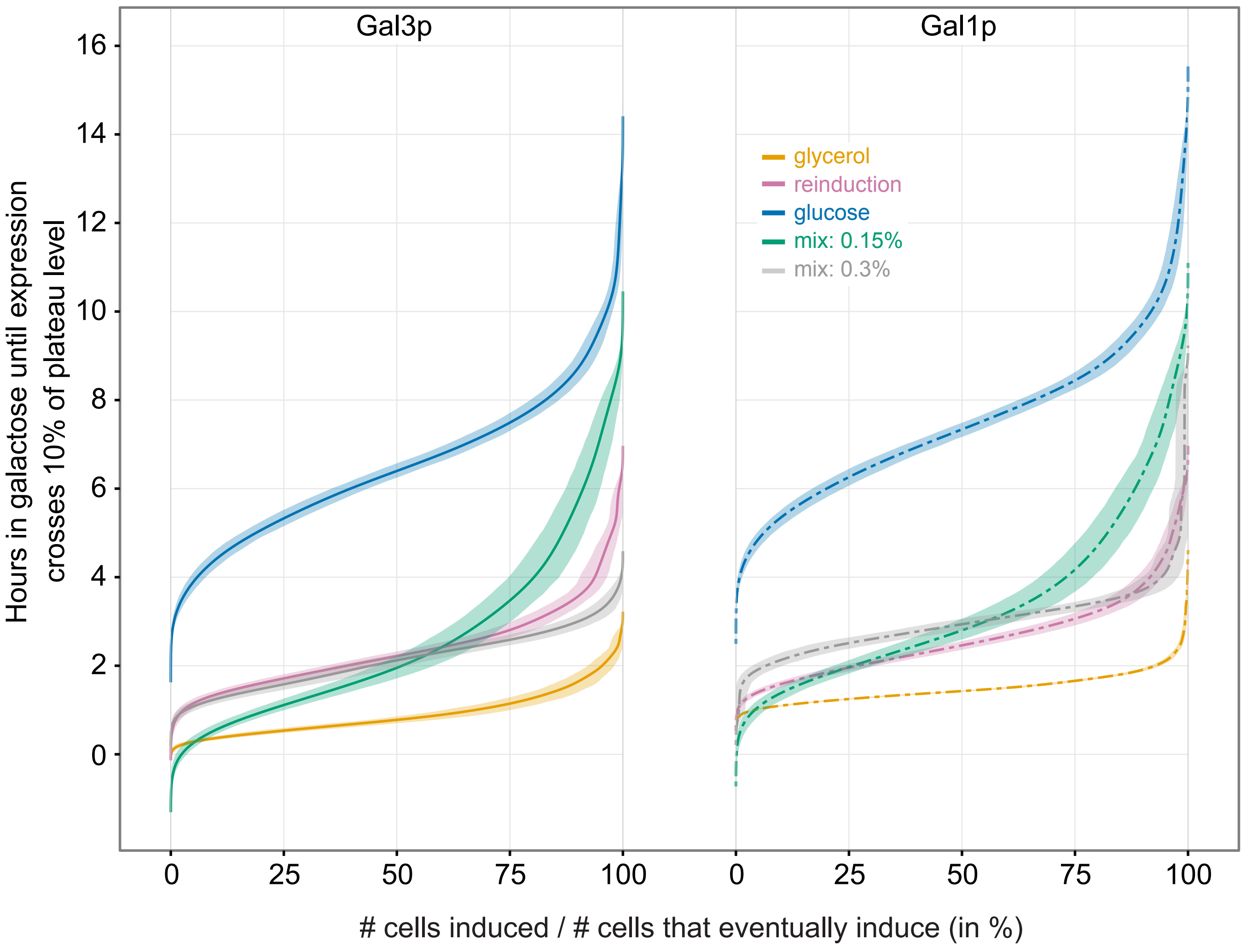

Appendix Figure S10. Empirical cumulative distributions of induction for each history condition for Gal3p and Gal1p. Only cells that started with expression levels below 10% and induced to at least 75% of the plateau level are included. Shaded areas denote 95% confidence intervals from bootstrapping.

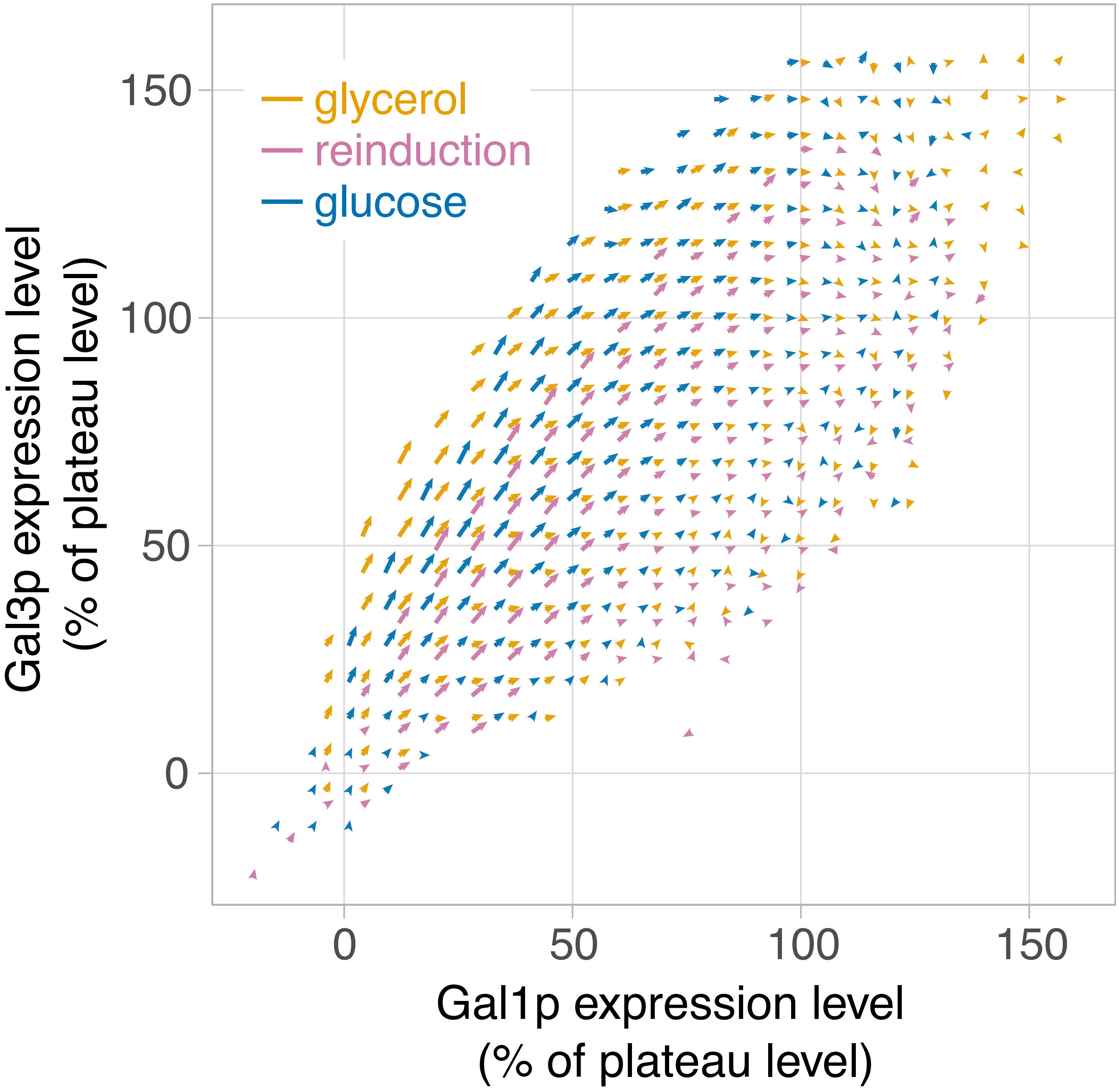

Appendix Figure S11. The vector fields for the initial three experimental conditions plotted together. Bins are slightly offset to separate the vectors and vector lengths are 1/3 the mean unsigned displacement to reduce crowding. Once cells are induced, they move in a consistent direction to plateau levels in all three experiments.

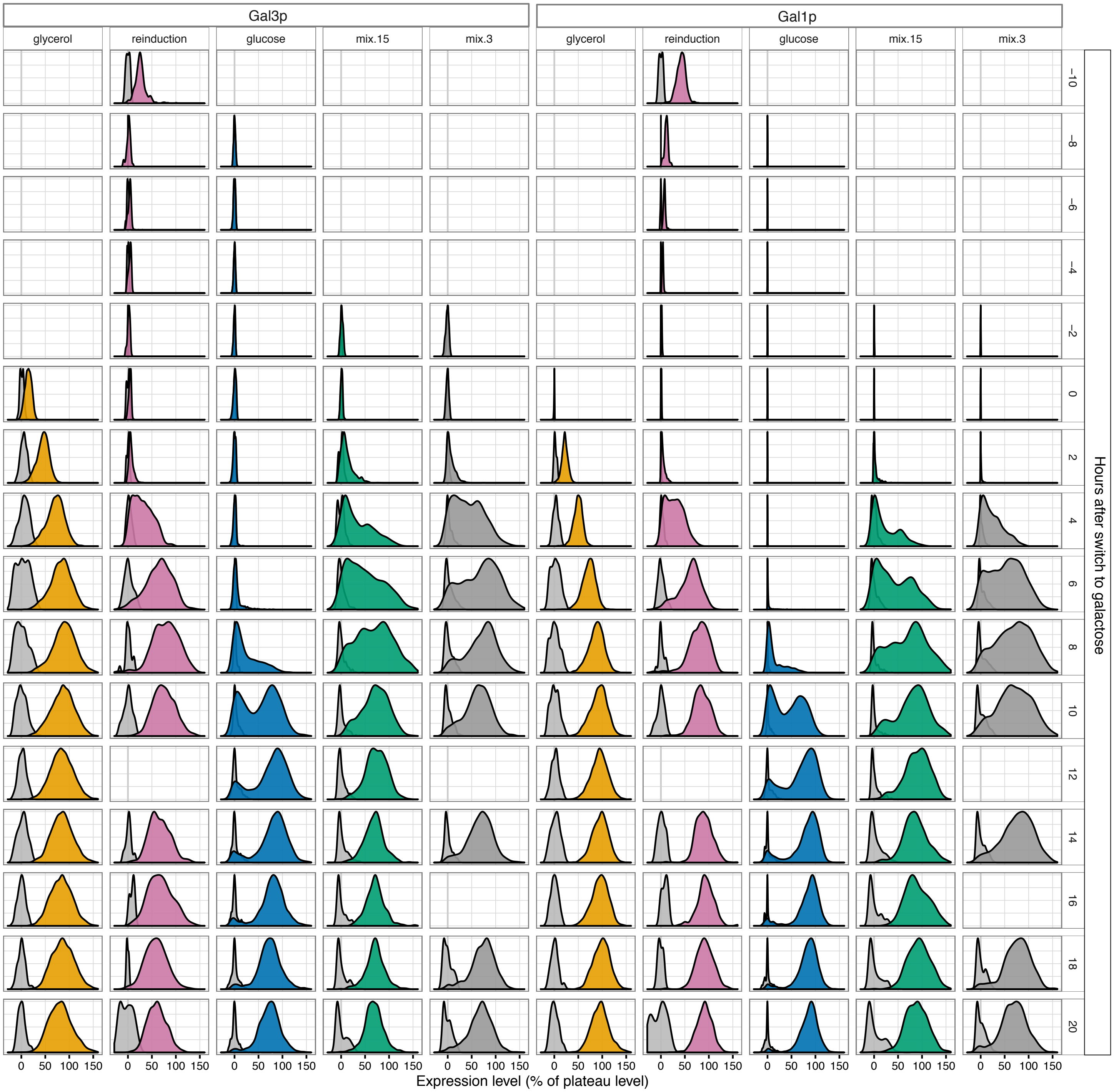

Appendix Figure S12. Population level depiction of galactose network induction. Empirical population densities for Gal3p and Gal1p induction levels in glycerol-history, reinduction, glucose, and the two glucose-galactose mix conditions. Grey densities represent control cells which have neither yECerulean or 2x-yECitrine. Empty blocks indicate frames in specific experiments where there were not enough cells to estimate a density reliably. In a few of the frames across the experiments the bright field images did not give a reliable segmentation.

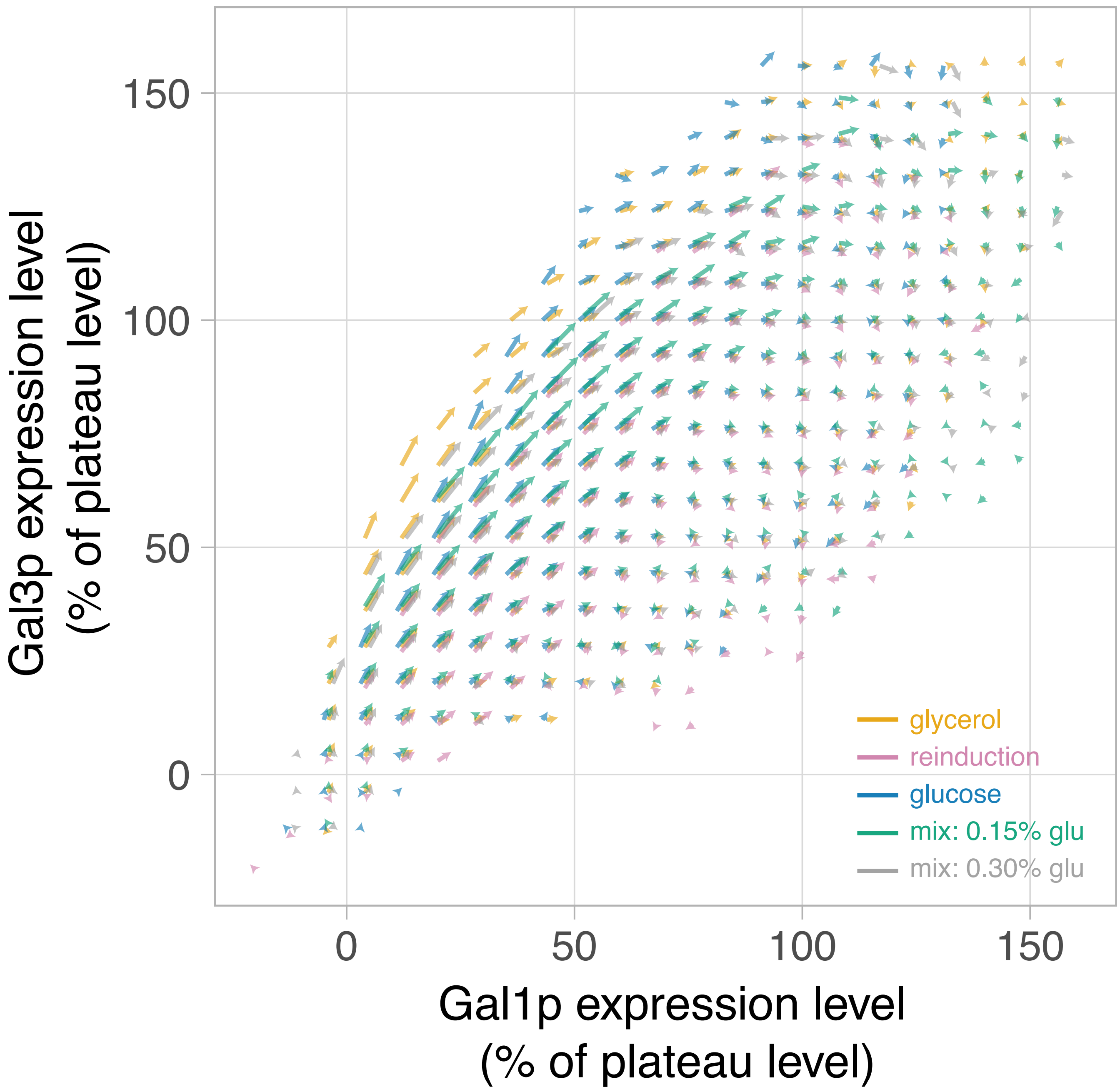

Appendix Figure S13. Vector fields for all five experiments overlaid
