# Supplementary material for: A living vector field reveals constraints on galactose network induction in yeast
Source: Mol Syst Biol. 2017 Jan 1;13(1):908. doi: 10.15252/msb.20167323 (PMC5293160; doi:10.15252/msb.20167323)
Supplement: Supplementary file 2 — Expanded View Figures PDF [file MSB-13-908-s002.pdf]

Expanded View Figures

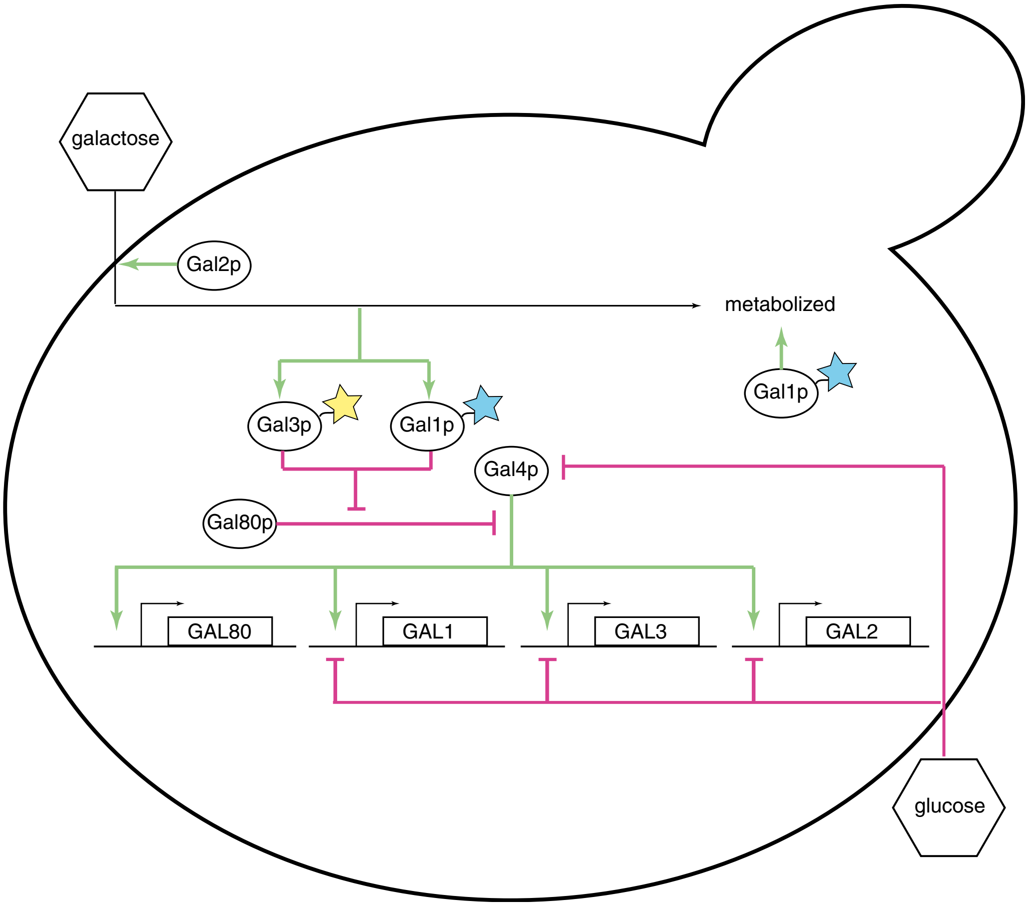

Figure EV1. A schematic of the regulatory portion of the galactose network in *Saccharomyces cerevisiae* including key targets of glucose repression.
Gal7p and Gal10p (not shown) help Gal1p metabolize galactose. Activating and repressing interactions are indicated by green and magenta, respectively. Molecular details of glucose repression are not shown.

© 2017 The Authors

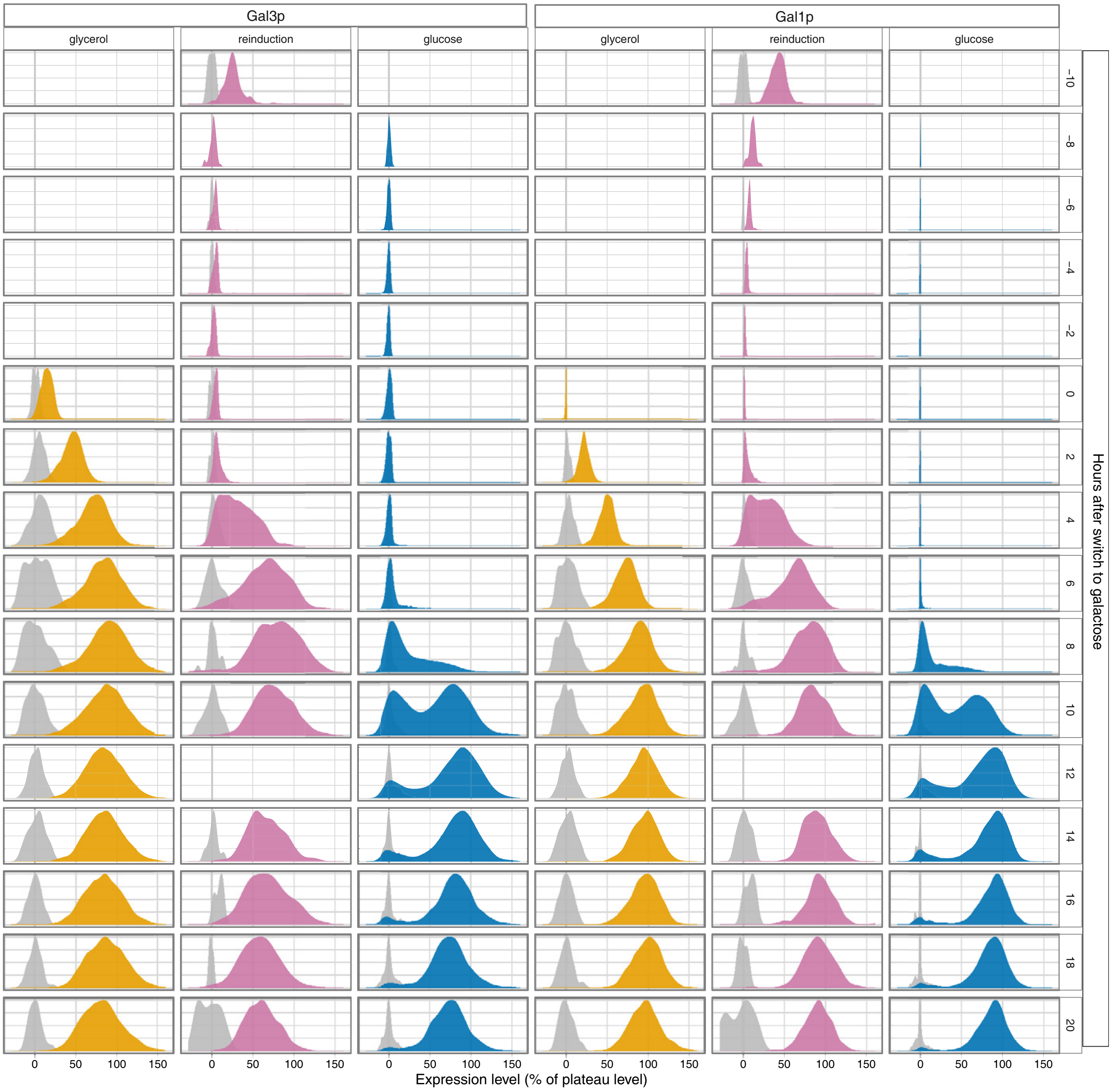

Figure EV2. Population-level depiction of galactose network induction.

Empirical population densities for Gal3p and Gal1p induction levels in glycerol-history, reinduction, and LTGR conditions. Grey densities represent control cells which have neither yECerulean nor 2x-yECitrine. Empty blocks in the reinduction experiment result from where there were not enough properly segmented cells to estimate a density reliably. In a few of the frames across the experiments, the bright-field images did not give a reliable segmentation.

 © 2017 The Authors

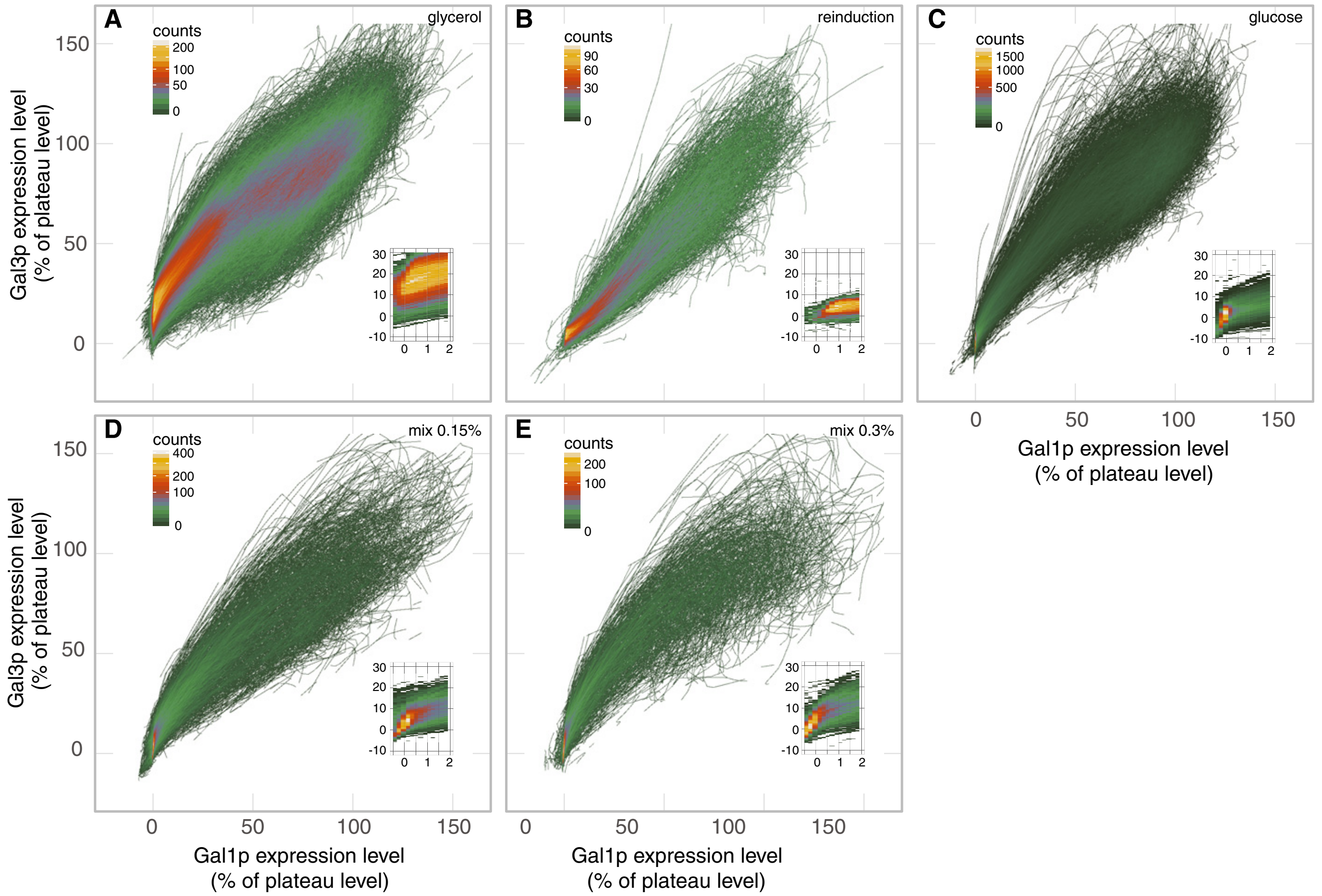

Figure EV3. Heat maps of the cell trajectories on the state space for the five conditions.

A–E Trajectory densities on the state space for glycerol (A), reinduction (B), glucose (C), 0.15% glucose mix (D), and 0.3% glucose mix (E). The color of a pixel indicates the number of trajectories that pass through it, color scales as marked. The insets depict the corner near (0%, 0%). The *x*-axes of the insets are Gal1p expression in terms of % of plateau level with grid lines at −0.5, 0, 0.5, 1, 1.5, and 2%. The *y*-axes are Gal3p expression in terms of % of plateau level with grid lines at −10, 0, 10, 20, and 30%. The glucose history conditions (glucose, 0.15% mix, 0.3% mix) are very similar in these plots but differ in timing which is not depicted here.

© 2017 The Authors
